# Medico-economic impact of thoracoscopy versus thoracotomy in lung cancer: multicentre randomised controlled trial (Lungsco01)

**DOI:** 10.1186/s12913-023-09962-y

**Published:** 2023-09-18

**Authors:** Anne-Laure Soilly, Ludwig Serge Aho Glélé, Alain Bernard, Halim Abou Hanna, Marc Filaire, Pierre Magdaleinat, Charles Marty-Ané, François Tronc, Renaud Grima, Jean-Marc Baste, Pascal-Alexandre Thomas, Bertrand Richard De Latour, Arnaud Pforr, Pierre-Benoît Pagès

**Affiliations:** 1 Direction of Clinical Research and Innovation, Clinical Research Unit-Methodological Support Network, CHU Dijon-Bourgogne, 21000 Dijon, France; 2 Department of Epidemiology, CHU Dijon, Dijon, France; 3 Department of Thoracic and Cardiovascular Surgery, CHU Dijon, Dijon, France; 4 Department of Thoracic Surgery and Endocrine Surgery, Centre Jean Perrin, Clermont Auvergne University, Clermont-Ferrand, France; 5 Department of Thoracic Surgery, AP-HP, Hôpital Cochin, Paris, France; 6 Department of Thoracic and Cardiovascular Surgery, Hôpital Arnaud de Villeneuve, CHU Montpellier, Montpellier, France; 7 Department of Thoracic Surgery, HCL, Hôpital Louis Pradel, Bron, France; 8 Department of Thoracic Surgery, CHU Rouen, Rouen, France; 9 Department of Thoracic Surgery, North University Hospital, Aix-Marseille University & APHM, Marseille, France; 10 Department of Thoracic and Cardiovascular Surgery, CHU Rennes, Rennes, France; 11 Department of Thoracic and Vascular Surgery, Avignon, Avignon, CH France

**Keywords:** Cost analysis, Cost-utility analysis, Thoracoscopy, Thoracotomy, Lung cancer surgery

## Abstract

**Background:**

Lungsco01 is the first study assessing the real benefits and the medico-economic impact of video-thoracoscopy versus open thoracotomy for non-small cell lung cancer in the French context.

**Methods:**

Two hundred and fifty nine adult patients from 10 French centres were randomised in this prospective multicentre randomised controlled trial, between July 29, 2016, and November 24, 2020. Survival from surgical intervention to day 30 and later was compared with the log-rank test. Total quality-adjusted-life-years (QALYs) were calculated using the EQ-5D-3L®. For medico-economic analyses at 30 days and at 3 months after surgery, resources consumed were valorised (€ 2018) from a hospital perspective. First, since mortality was infrequent and not different between the two arms, cost-minimisation analyses were performed considering only the cost differential. Second, based on complete cases on QALYs, cost-utility analyses were performed taking into account cost and QALY differential. Acceptability curves and the 95% confidence intervals for the incremental ratios were then obtained using the non-parametric bootstrap method (10,000 replications). Sensitivity analyses were performed using multiple imputations with the chained equation method.

**Results:**

The average cumulative costs of thoracotomy were lower than those of video-thoracoscopy at 30 days (€9,730 (SD = 3,597) *vs.* €11,290 (SD = 4,729)) and at 3 months (€9,863 (SD = 3,508) *vs.* €11,912 (SD = 5,159)). In the cost-utility analyses, the incremental cost-utility ratio was €19,162 per additional QALY gained at 30 days (€36,733 at 3 months). The acceptability curve revealed a 64% probability of efficiency at 30 days for video-thoracoscopy, at a widely-accepted willingness-to-pay threshold of €25,000 (34% at 3 months). Ratios increased after multiple imputations, implying a higher cost for video-thoracoscopy for an additional QALY gain (ratios: €26,015 at 30 days, €42,779 at 3 months).

**Conclusions:**

Given our results, the economic efficiency of video-thoracoscopy at 30 days remains fragile at a willingness-to-pay threshold of €25,000/QALY. The economic efficiency is not established beyond that time horizon. The acceptability curves given will allow decision-makers to judge the probability of efficiency of this technology at other willingness-to-pay thresholds.

**Trial registration:**

NCT02502318.

## Background

Lung cancer (LC) remains a major health problem with an estimated 130,180 deaths in 2022 in the US, which makes it the leading cause of cancer death in both sexes [1]. For early-stage LC, surgery remains the cornerstone of management and results in better overall survival [2]. Lobectomy with mediastinal lymph node dissection is therefore recommended in order to limit the risk of local recurrence [3]. However, lobectomy is associated with a high proportion of postoperative complications, especially respiratory complications, ranging from 12% in the analysis of the American Society of Thoracic Surgeons database, around 15% in the French National Epithor database, to 30% in a recent study using the SEER database [4–6]. Moreover, as reported by Stéphan et al., postoperative respiratory complications are associated with increases in mortality and length of stay in the intensive care or surgical ward, which has an effect on health system spending [7].

During the last decade, the use of video-assisted thoracoscopic surgery (VATS) lobectomy for LC has grown considerably. In France, the use of VATS jumped from 12% of lobectomies in 2012 to 48% in 2017 [8].

Recommendations from the American College of Chest Physicians in 2013 and the European Society of Medical oncology in 2014 suggest that VATS lobectomy be performed depending on the experience of the surgeon and for tumors of any stage [2, 3]. These recommendations are based on meta-analyses from the last decade, which indicated that VATS resulted in shorter hospital stays and fewer postoperative complications such as atelectasis or pneumonia [9–14]. However, the data used to formulate these recommendations were of poor quality. There are few randomized controlled trials (RCTs) in the literature evaluating the benefits of VATS compared to open thoracotomy for LC. In fact, only Bendixen et al., in a trial involving 206 patients, found that there was a benefit regarding postoperative pain and quality of life during the first year of follow-up in favor of VATS versus open thoracotomy [15].

To date, only four RCTs comparing complications and overall survival following VATS lobectomy or open thoracotomy for the treatment of lung cancer have been published in English [16–19]. The authors did not find VATS to be more beneficial than open thoracotomy except for intraoperative blood loss and median time of surgery. There were no benefits in terms of postoperative complications, mortality or length of hospital stay.

More recently, Bendixen et al. reported the results of the cost-utility analysis of VATS lobectomy performing a RCT including 103 patients in each group. They highlighted that VATS lobectomy was a cost-effective alternative to open thoracotomy for stage I LC [20].

The medico-economic literature on this subject is poor, which is why a medico-economic study is now needed to determine whether the costs induced by VATS are offset by the reduction in postoperative complications. This paper presents the results of a large RCT assessing the medico-economic impact of VATS lobectomy when compared with open thoracotomy in the French context.

The primary aim of this study was to evaluate the cost-effectiveness and the cost-utility impacts of VATS lobectomy when compared with open thoracotomy for the management of non-small cell lung cancer (NSCLC). These medico-economic analyses were performed at 30 days after surgery. The secondary aim was to evaluate the cost-effectiveness and cost-utility impacts at 3 months after surgery.

## Material and methods

Lungsco01 is an open two-arm parallel RCT comparing lobectomy or segmentectomy performed by VATS with lobectomy or segmentectomy using thoracotomy for the treatment of LC. As specified in the published study protocol, the study involved French thoracic surgery departments that had already performed more than 50 VATS lobectomies [21]. The number of patients in each of the treatment groups was calculated and planned to be equal (300 patients in each arm), with a ratio of 1:1 and a stratification by centre since the different practices of each team may have an impact on the judgement criterion [21]. The list of randomised patients was divided into blocks of 12 to obtain balanced groups. Randomisation was available after patients had met the inclusion criteria [21].

### Study population

The study population included patients with proven or suspected NSCLC which could be treated by lobectomy or segmentectomy performed by VATS or lobectomy or segmentectomy using thoracotomy. Inclusion criteria and exclusion criteria were described in the published protocol [21].

### Procedures

There were two potential approaches for lobectomy or segmentectomy using video-thoracoscopy (VATS) and two potential types of thoracotomy: posterolateral thoracotomy with muscle sparing or lateral thoracotomy [18, 21]. At the start of the study, each surgical team chose one of the two approaches according to their experience. The chosen approach was the one to be used throughout the trial.

### Post-operative care

Postoperatively, whether after VATS or thoracotomy, analgesia (morphine) was delivered via the epidural catheter or paravertebral catheter or intravenously. All patients had respiratory and motor physiotherapy immediately after the surgery at least twice a day during the hospital stay. All patients had a nasal cannula. Saline aerosols were prescribed if the patient had difficulty expectorating. Thrombophlebitis was prevented by stockings and anticoagulants (managed according to the usual practice of each centre) [21].

### Outcomes

As part of the cost-effectiveness analyses initially planned, incremental cost-effectiveness ratios (ICER) were to be calculated taking into account the cost differential and the survival differential between the two groups. The ratios would have been expressed as the additional cost per life-year gained using the innovative technique (VATS) compared with the reference technique (thoracotomy), at 30 days and at 3 months after surgery. However, since death was infrequent and not different at these time horizons, only the cost differential was considered. This led us to perform cost-minimisation analyses [22], which corresponds to a sub-category of cost-effectiveness analyses.

As part of the cost-utility analyses, incremental cost-utility ratios (ICER) were estimated by relating the cost differential to the average differential in quality adjusted life years (QALYs) between the two strategies, at 30 days and at 3 months. The ICER represent the additional cost necessary to gain one additional QALY using the innovative technique (VATS) compared with the reference technique (thoracotomy).

### Data collection

All the clinical data, resources consumed and responses to the EQ-5D-3L® questionnaire were collected prospectively via an electronic Case Report Form (e-CRF).

### Cost estimations

The costs of VATS and thoracotomy were estimated for each patient for the 30 first days and the 3 first months after surgery. Costs were estimated from the hospital perspective given the available data on resources consumed (hospital stays). They included: (i) the production costs of the initial stays (including surgery); (ii) the production costs of all re-hospitalisations related to post-surgical complications; and (iii) the production costs of all admissions to rehabilitation care.

In the first step, diagnosis-related groups (DRGs) corresponding to all patient stays were identified in accordance with the rules for the classification of stays in France. Thus, the DRG for each initial stay and each re-hospitalisation for complications were assigned according to comorbidities, the reason for admission, the complications arising during the stay (and possible re-intervention), and length of stay (LOS). DRGs for stays in rehabilitation care were assigned using patient comorbidities, the reason for admission, the patient’s age, and time between the date of surgery and admission to rehabilitation care.

In the second step, a production cost was applied to each stay from the *Etude Nationale des Coûts à méthodologie Commune* (ENCC), a national survey of production costs per DRG estimated from a sample of representative hospitals in France. These production costs are broken down by expenditure item and type of service attended, and published annually by the French Agency for Information on Hospital Care (ATIH) [23, 24]. The last year available in the ENCC at the time of analyses was 2018, which determined our reference year for costing.

Especially for initial stays (including surgery), using the ‘*adjusted-DRG*’ method made it possible to replace the cost of the operating room issued from the ENCC with the actual real production costs of surgery estimated by micro-costing [25]. These real costs (VATS: €3,870.49; Thoracotomy: €2,455.58) were estimated in a previous publication from a sample of fifty patients from the Lungsco01 trial, from July 2015 to July 2016 [26]. For the needs of the paper, the costs have been updated to 2018 euros using the annual harmonized Consumer Price Index (CPI—base 100 in 2015) from the European Classification of Individual Consumption by Purpose (ECOICOP nomenclature – Health division—06.3.0.1.1 Hospital services—France) [27]. Finally, since the ENCC database contains the national LOS per DRG, an average daily cost was calculated in order to reconstitute a cost for the rest of the stay weighted by the LOS for each patient [28]. No discounting has been undertaken on costs in the absence of a time horizon greater than 12 months.

### Utility measure

QALYs were evaluated using collected responses to the EQ-5D-3L® generic questionnaire completed pre-operatively, 3 days after surgery, during the day-30 visit and during the 3-month visit after surgery [29]. For each patient, final QALYs at 30 days and at 3 months were calculated by taking into account the periods between two administration times, as recommended [30]. No discounting has been undertaken in the absence of a time horizon greater than 12 months.

### Statistical analyses

Analyses were performed on an intention-to-treat basis. Qualitative variables were compared using the χ^2^ test (or Fisher’s test if the expected values were < 5). Continuous variables were compared using Student’s t-test, which was also used for cost comparisons since non-parametric statistical tests are not recommended [31]. We used the difference in risk of death and logistic regression model to compare deaths at 30 days and 90 days, allowing estimation of odds ratios with their 95% confidence intervals.

The cost-utility analyses at 30 days and at 3 months were performed on cases with complete QALYs (no missing data). Then, a non-parametric bootstrap method (10,000 replications) was used in order to study the uncertainty associated with the sample and to construct a 95% confidence interval (95% CI) for the ICERs. This was followed by the construction of a Cost-Utility plane (CU-plane) and an acceptability curve from the bootstrap and the 95% CI of the ICERs. This curve is used to represent the probability that the VATS strategy is efficient when compared to the thoracotomy strategy (y-axis) as a function of different possible values of society’s Willingness-To-Pay (WTP) for an additional health unit (x-axis). A sensitivity analysis was performed using multiple imputation by chained equation method in order to take into account patients with missing data [31]. As done previously, this was followed by bootstrap analyses (10,000 replications) to construct the 95% CI for the ICERs. For the multiple imputation procedure, the following variables were used in the imputed datasets in order to improve the accuracy of imputed data: age; gender; performance status; smoking status (smoker/ex-smoker/non-smoker); body mass index; responses to the 5 items of the EQ-5D-3L® preoperatively, 3 days, 30 days and 3 months after surgery; and all hospital cost variables (cost of initial stay, re-hospitalisations and admissions in rehabilitation). The number of imputations was 10. The characteristics of patients with and without missing data were compared (results not shown). The stability between results of the complete cases and imputed data analysis were described.

The version 9.4 SAS was used for the analyses. The threshold for significance was set at 0.05.

## Results

**Fig. 1:**
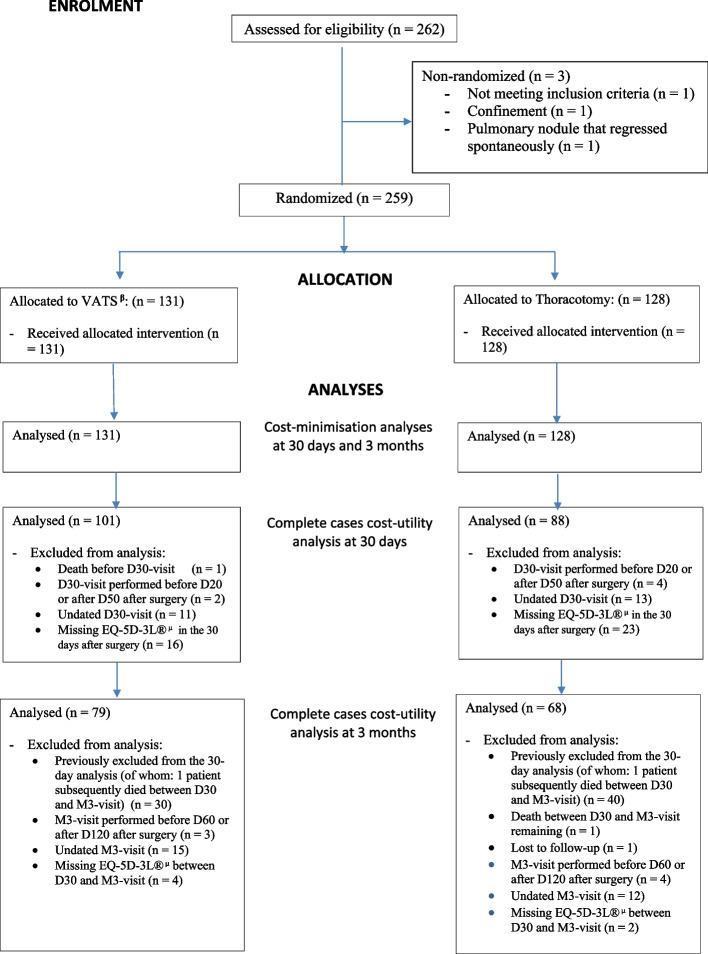
Flowchart. This flowchart describes the enrolment of patients in this study, their allocation in the VATS or thoracotomy group, and finally the selection of patients for complete cases analyses. Particularly for the cost-utility analyses, complete cases for Quality-Adjusted Life-Years (QALY) were considered ^β^Video-Assisted Thoracoscopic Surgery. ^µ^EuroQol-5 Dimensions-3 Levels questionnaire

Two hundred and sixty-two patients were assessed for eligibility and signed consent to participate in the study from July 29, 2016 to November 24, 2020. One hundred and thirty-one patients were randomised to the VATS group, 128 to the thoracotomy group, and 3 were not randomized (Fig. 1). Ten centres participated in the study.

### Patient characteristics

There was no significant difference in the baseline clinical characteristics between the two groups (Table 1). Intervention, post-operative respiratory complications and other major complications, re-hospitalisations, and QALYs assessed at each administration time are described (Table 2). The only significant differences between the two groups were the mean duration of the procedure (4.36 h for VATS *vs.* 3.73 for thoracotomy; *p*-value < 0.001), and the percentage of patients re-hospitalized between the 30-day and the 3-month visits (8.39% *vs.* 2.34%; *p*-value = 0.05). There was no significant difference in overall survival between the two groups at 30 days after the surgery (99.25% [95% CI: 94.82 – 99.89] for VATS *vs.* 99.22% [95% CI: 94.58 – 99.89] for thoracotomy), or at 3 months after the surgery (81.21% [95% CI: 45.15 – 94.70] for VATS *vs.* 79.37% [95% CI: 41.27 – 94.15] for thoracotomy). There was no significant difference in the risk of death between the two groups. The difference in risk of death at 30 days was -0.035 after thoracotomy compared to after VATS (95% CI: -0.09 to 0.02; *p*-value = 0.2155). The difference in risk of death at 3 months was -0.004 after thoracotomy compared to after VATS (95% CI: -0.07 to 0.06; *p*-value = 0.8929). For 30-day mortality, the odds ratio was 0.5 (95% CI: 0.17 - 1.52). For 90-day mortality, the odds ratio was 0.94 (95% CI: 0.37–2.4).

**Table 1:** Baseline clinical characteristics

**Characteristics**	**VATS**^a^**(*N* = 131)**	**Thoracotomy (*N* = 128)**	**Total (*N* = 259)**
Gender, no. (%)			
Male	82 (62.60)	76 (59.38)	158 (61.00)
Female	49 (37.40)	52 (40.61)	101 (39.00)
Age, mean (SD), years	65.50 (8.38)	63.40 (10.24)	64.46 (29.00)
Body-mass index, mean (SD), kg/m^2^	26.28 (4.63)	26.84 (5.43)	26.56 (5.04)
Performance status, no. (%)			
0	94 (71.76)	89 (69.53)	183 (70.66)
1	37 (28.24)	39 (30.47)	76 (29.34)
Tobacco use, no. (%)			
Smoker	39 (29.77)	38 (29.69)	77 (29.73)
Ex-smoker	66 (50.38)	69 (53.91)	135 (52.12)
Non-smoker	26 (19.85)	21 (16.41)	47 (18.15)
Medical history, no. (%)			
Yes	111 (84.73)	112 (87.50)	223 (87.80)
No	17 (12.98)	14 (10.94)	31 (12.20)
Surgical history, no. (%)			
Yes	8 (6.11)	6 (4.69)	14 (5.51)
No	120 (91.60)	120 (93.75)	240 (94.49)
Location of tumor, no. (%)			
Upper right lobe	43 (32.82)	43 (33.86)	86 (35.54)
Upper left lobe	36 (27.48)	29 (22.83)	65 (26.86)
Middle lobe	6 (4.58)	3 (2.36)	9 (3.72)
Lower right lobe	20 (15.27)	28 (22.05)	48 (19.83)
Lower left lobe	18 (13.74)	16 (12.60)	34 (14.05)
Histology, no. (%)			
Squamous cell carcinoma	24 (18.32)	25 (19.69)	49 (20.42)
Adenocarcinoma	83 (63.36)	76 (59.84)	159 (66.25)
Adenosquamous carcinoma	1 (0.76)	1 (0.79)	2 (0.83)
Large cell or large cell neuroendocrine carcinoma	3 (2.29)	2 (1.57)	5 (2.08)
Carcinoma with pleomorphic or sarcomatoid elements	0 (0.00)	0 (0.00)	0 (0.00)
Carcinoid tumor	3 (2.29)	3 (2.36)	6 (2.50)
Other histological type	7 (5.34)	12 (4.72)	19 (7.92)
p T, no. (%)			
T1a	18 (13.74)	17 (13.39)	35 (15.91)
T1b	19 (14.50)	26 (20.47)	45 (20.45)
T1c	11 (8.40)	7 (11.81)	18 (8.18)
T2a	47 (35.88)	43 (33.86)	90 (40.91)
T2b	6 (4.58)	5 (3.94)	11 (5.00)
T3	11 (8.40)	8 (6.30)	19 (8.64)
T4	1 (0.76)	1 (0.79)	2 (0.91)
p N, no. (%)			
N0	95 (72.52)	95 (74.80)	190 (85.97)
N1	10 (7.63)	8 (6.30)	18 (8.14)
N2	8 (6.11)	5 (3.94)	13 (5.88)
N3	0 (0.00)	0 (0.00)	0 (0.00)
Stage, no. (%)			
IA	41 (36.94)	40 (40.40)	81 (39.51)
IB	39 (35.14)	36 (36.36)	75 (36.59)
IIA	9 (8.11)	11 (11.11)	20 (9.76)
IIB	8 (7.21)	5 (5.05)	13 (6.34)
IIIA	11 (9.91)	5 (5.05)	16 (7.80)
Lung resection side, no. (%) of patients			
Right	70 (53.44)	76 (59.38)	146 (59.59)
Left	54 (41.22)	45 (35.16)	99 (40.41)
Segmentectomy, no. (%) of patients			
Yes	11 (8.40)	14 (10.94)	25 (10.37)
VEMS^b^, mean (SD)	95.88 (17.69)	95.28 (18.75)	95.59 (18.18)

^a^*VATS* Video-Assisted Thoracoscopic Surgery

^b^*VEMS* Maximum Expiratory Volum per Second

**Table 2:** Intervention, complications, rehospitalisations and QALYs at different times of collection

	**VATS**^a^** (*N* = 131)**	**Thoracotomy (*N* = 128)**	***p*-value**
**Intervention**			
Type of thoracotomy, no. (%) of patients			
Posterolateral thoracotomy with muscle sparing	-	46 (35.94)	
Lateral thoracotomy	-	72 (56.25)	
Intraoperative complications, no. (%) of patients			
Yes	24 (18.32)	12 (9.38)	**0.037**
No	101 (77.10)	110 (85.94)	
Conversion to thoracotomy, no. (%) of patients	13 (9.92)	-	
Duration mean (SD), hours	4.36 (1.00)	3.73 (0.84)	** < 0.001**
**Post-surgery**			
Total length of hospital stay, mean (SD), days	7.20 (3.72)	7.13 (2.85)	0.873
Postoperative respiratory complications^b^, no. (%) of patients	2 (1.5%)	3 (2%)	0.681
**Re-hospitalisations**			
**From hospital discharge to 30-day visit:**			
Re-hospitalisation, no. (%) of patients	6 (4.58)	4 (3.13)	1.000
Rehabilitation care^c^, no. (%) of patients	9 (6.87)	8 (6.25)	0.619
**From 30-day to 3-month visit:**			
Re-hospitalization, no. (%) of patients	11 (8.39)	3 (2.34)	1.000
Rehabilitation care^d^, no. (%) of patients	5 (3.82)	0 (0.00)	0.060
**QALYs at each time collection**			
**Preoperatively**			
no. (%) of patients without EQ-5D-3L®^e^ missing data	115 (87.78)	117 (91.40)	
average number of QALYs^f^ (SD)	0.83 (0.20)	0.78 (0.26)	0.082
**At day 3 post-surgery**			
no. (%) of patients without EQ-5D-3L®^e^ missing data	111 (84.73)	103 (80.47)	
average number of QALYs^f^ (SD)	0.66 (0.31)	0.58 (0.28)	0.053
**At day 30 post-surgery**			
no. (%) of patients without EQ-5D-3L®^e^ missing data	112 (85.49)	108 (84.37)	
average number of QALYs^f^ (SD)	0.80 (0.21)	0.74 (0.26)	0.074
**At 3 months post-surgery**			
no. (%) of patients without EQ-5D-3L®^e^ missing data	99 (75.57)	89 (69.53)	
average number of QALYs^f^ (SD)	0.79 (0.27)	0.76 (0.30)	0.449

^a^Video-Assisted Thoracoscopic Surgery

^b^Postoperative respiratory complications include at least one of the following elements: the use Non-invasive ventilation or atelectasis or pneumonia or mechanical ventilation or reintubation or acute respiratory distress syndrome

^c^Rehabilitation care in the 30 days after hospital discharge include admission in rest home or in rehabilitation

^d^Rehabilitation care between 30-days visit and 3-months visit include admission in convalescent home or in a medium stay structure

^e^EuroQol-5 Dimensions-3 Levels questionnaire

^f^Quality Adjusted Life Years

The QALYs assessed at each point of administration were slightly higher for VATS compared to thoracotomy, but the difference was not significant (Table 2).

### Cost-minimisation analyses

There was a difference in average cumulative costs at 30 days of €1,560 (95% CI: €531 to €2,587) between the groups, with significantly higher costs in the VATS group than in the thoracotomy group (*p*-value = 0.003). There was a difference in average cumulative costs at 3 months of €2,049 (95% CI: €971 to €3,127) between the groups, with a significantly higher costs in the VATS group than in the thoracotomy group (*p*-value =  < 0.001). In both analyses, the costs attributed to VATS were higher than those of thoracotomy, mainly due to the cost of the initial stay (€10,670 in the VATS group *vs.* €9,213 in the thoracotomy group; *p*-value =  < 0.001). The detailed cost results are presented in Table 3.

**Table 3:** Comparison of the cumulative cost differential between the two strategies (cost-minimisation analyses)

	**VATS**^a^			**THORACOTOMY**				
	**N**	**Mean** A	**Median**	**N**	**Mean** B	**Median**	**Difference of means** A-B	***p*-value**
		(SD)	[Q1-Q3]		(SD)	[Q1-Q3]	[95% CI]	
**Initial stay**								
Costs (€ 2018)	131	10,670	9,853	128	9,213	8,442	1,457	< 0.001
		(3,941)	[8,858–11,844]		(2,807)	[7,447–10,433]	[621–2,294]	
**From hospital discharge to the 30-day visit**								
Costs of re-hospitalisations (€ 2018)	6	4,205	2,716	4	4,116	3,793	89	0.959
		(2,816)	[2,562–6,106]		(2,211)	[2,523–5,708]	[-3,967 ; 3,789]	
Costs of rehabilitation care (€ 2018)^b^	9	6,222	6,222	8	6,222	6,222	-	-
		(0)	[6,222–6,222]		(0)	[6,222–6,222]		
**From the 30 days-visit to the 3 months-visit**								
Costs of re-hospitalisations (€ 2018)	10^c^	3,246	2,324	3	3,168	3,003	78	0.964
		(2,712)	[1,751–3,814]		(1,566)	[1,692–4,810]	[-3,606 ; 3,762]	
Costs of rehabilitation care (€ 2018)^b^	5	6,222	6,222	0	-	-	6,222	< 0.001
		(0)	[6,222–6,222]		-	-	[6,222–6,222]	
**Cumulative costs at 30 days (€ 2018)**^d^	131	**11,290**	9,981	128	**9,730**	8,442	**1,560**	0.003
		(4,729)	[8,858–11,844]		(3,597)	[7,447–10,433]	[531–2,587]	
**Cumulative costs at 3 months (€ 2018)**^e^	131	**11,912**	10,849	128	**9,863**	8,442	**2,049**	< 0.001
		(5,159)	[8,858–12,543]		(3,508)	[7,447–10,931]	[971- 3,127]	

^a^Video-Assisted Thoracoscopic Surgery

^b^All costs of rehabilitation care include costs of rest home, admission in rehabilitation, convalescent home and medium stay structure. They were calculated from a same assigned DRG (0403B1: “*Malignant tumours of the respiratory system, phy score* ≥ *5, cog score* ≤ *2, level 1”*; € 6,222.20) according to the indications of the medical information department of the University Hospital of Dijon

^c^One out of the 11 re-hospitalisations could not be costed due to a lack of data to identify the DRG

^d^Cumulative costs at 30 days were calculated by adding all the hospital costs from the initial stay to the 30-days visit (costs of the initial stay + costs related to all rehospitalisations and rehabilitation care over the 30 days after surgery)

^e^Cumulative costs at 3 months were calculated by adding all the hospital costs from the initial stay to the 3-months visit (costs of the initial stay + costs related to all rehospitalisations and rehabilitation care over the 30 days after surgery + costs related to all rehospitalisations and rehabilitation care over the 3 months after surgery)

### Cost-utility analyses

**Fig. 2:**
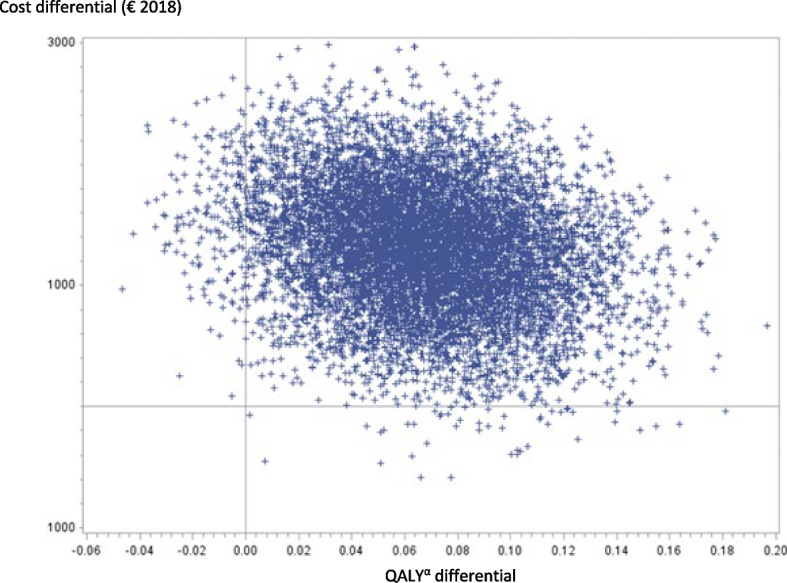
Cost-utility plane based on 10,000 bootstrapped replicates (at 30 days). The cost-utility plane (CU-plane) of the ICER provides a visual representation of the new strategy compared to the baseline strategy. It is constructed from the 10,000 samples generated by the bootstrap. A total of 97.61% of the bootstrapped ICER were located in the northeast quadrant of the CU-plane, meaning higher costs and more QALYs following VATS

**Fig. 3:**
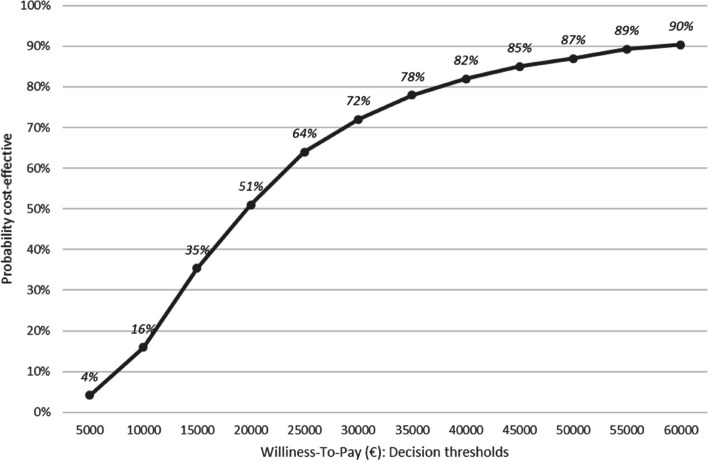
Acceptability curve for the choice of strategy (at 30 days). This curve makes it possible to evaluate the probability that the VATS strategy will be cost-effective at 30 days according to several willingness-to-pay (WTP) thresholds. It is based on the 10,000 samples generated by the bootstrap analysis. At each value of the WTP threshold (x-axis), the curve gives the proportion of samples for which the ICER ratio is below this WTP value. This proportion (y-axis) reflects the probability for which the VATS strategy is more efficient than the thoracotomy strategy at the WTP value

The cost-utility analysis at 30 days was performed on 189 patients (101 VATS and 88 thoracotomies), after excluding in this order: (i) patients who died before the D30-visit; (ii) patients for whom the D-30 visit was performed much earlier or later than 30 days; (iii) patients with an undated D30-visit; (iv) patients with missing data for the EQ-5D-3L® (Fig. 1). As shown in Table 4, there was a difference in average cumulative costs at 30 days of €1,303 (95% CI: €331 to €2,275), with significantly higher average cumulative costs for the VATS group (*p*-value = 0.009). There was a difference in the average number of final QALYs at 30 days of 0.068 (95% CI: 0.0036 to 0.1323), significantly in favour of the VATS group (*p*-value = 0.038). This resulted in an ICER of €19,162 per additional QALY gained. The bootstrap analysis resulted in a mean ICER of €126,304 (95% CI:-€138,157 to €390,765). A total of 97.61% of the bootstrapped cost-utility pairs were located in the northeast quadrant of the CU-plane, meaning higher costs and more QALYs following VATS (Fig. 2).The acceptability curve (Fig. 3) revealed a 64% probability of efficiency at 30 days for VATS at a widely-accepted WTP threshold of €25,000 per QALY gained [32]. The results of the cost-utility analysis by multiple imputation (*N* = 254 patients) revealed an increase in the ICER (ICER: €26,015 per QALY) (Table 4) due to a slight increase in the average cost differential and a slight decrease in the QALY differential after imputation.

**Table 4:** Cost-utility analysis, 30 days

	**VATS**^a^			**THORACOTOMY**					
	**N**	**Mean** A	**Median**	**N**	**Mean** B	**Median**	**Difference of means** A-B	***p*-value**	**ICER**^c^ €/QALY
		(SD)	[Q1-Q3]		(SD)	[Q1-Q3]	[95% IC]		
**Complete cases**									
Cumulative costs (€ 2018)	101	11,116	10,849	88	9,813	8,506	**1,303**	0.009	**19,162**
		(3,238)	[8,858–11,844]		(3,534)	[7,447–10,433]	[331 - 2,275]		
Final QALYs^b^ (year)	101	0.739	0.780	88	0.671	0.738	**0.068**	0.038	
		(0.214)	[0.585–0.901]		(0.234)	[0.540–0.862]	[0.0036–0.1323]		
Non-parametric bootstrap^e^									126,304
									95% CI: [-138,157 to 390,765]
**Imputed data**^d^									
Cumulative costs (€ 2018)	129	11,095	9,981	125	9,711	8,442	**1,384**	0.049	**26,015**
		(4,140)	[8,858–11,844]		(3,609)	[7,447–10,433]	[423–2,345]		
Final QALYs^b^ (year)	129	0.733	0.784	125	0.679	0.738	**0.053**	0.061	
		(0.2225)	[0.593–0.895]		(0.228)	[0.581–0.855]	[0.0025–0,1089]		
Non-parametric bootstrap^e^									11,995
									95% CI: [-5,613 to 29,603]

^a^Video-Assisted Thoracoscopic Surgery

^b^Quality-Adjusted-Life-Years

^c^Incremental Cost-utility Ratio (noted *ICER*) calculated by relating the difference in mean costs to the difference in mean utilities (Final QALYs) between the two groups. This ratio is interpreted as the additional cost necessary to gain one additional QALY using the innovative technique (VATS) compared to the reference technique (thoracotomy)

^d^Imputation excluded patients dead and patients who left the study early (lost to follow-up)

^e^Bootstrap is based on 10,000 replications

**Fig. 4:**
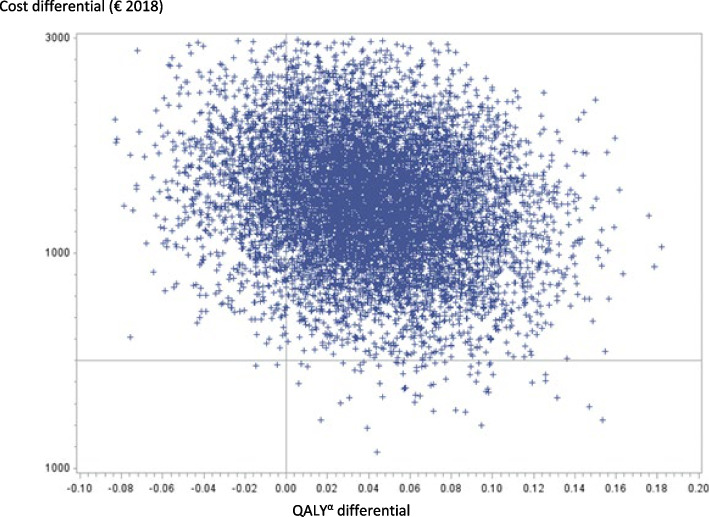
Cost-utility plane based on 10,000 bootstrapped replicates (at 3 months). The cost-utility plane (CU-plane) of the ICER provides a visual representation of the new strategy compared to the baseline strategy. It is constructed from the 10,000 samples generated by the bootstrap. A total of 86.14% of the bootstrapped ICER were located in the northeast quadrant of the CU-plane, meaning higher costs and more QALYs following VATS

**Fig. 5:**
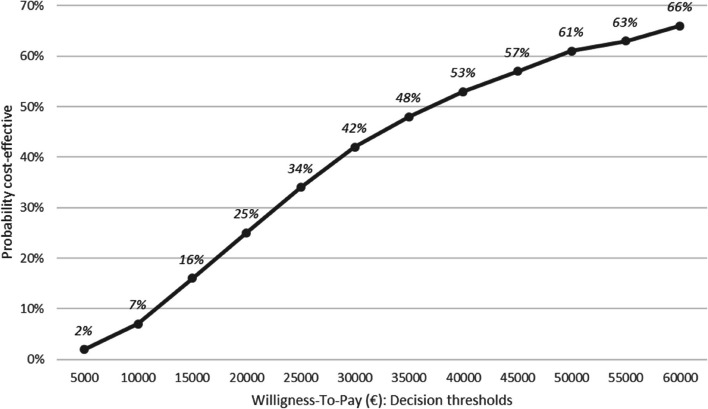
Acceptability curve for the choice of strategy (at 3 months). Acceptability curve for the choice of strategy. This curve makes it possible to evaluate the probability that the VATS strategy will be cost-effective at 3 months according to several willingness-to-pay (WTP) thresholds. It is based on the 10,000 samples generated by the bootstrap analysis. At each value of the WTP threshold (x-axis), the curve gives the proportion of samples for which the ICER ratio is below this WTP value. This proportion (y-axis) reflects the probability for which the VATS strategy is more efficient than the thoracotomy strategy at the WTP value

The cost-utility analysis at 3 months was carried out on 147 patients (79 VATS and 68 thoracotomies) (Fig. 1) after excluding, in this order: (i) patients previously excluded from the complete cases cost-utility analysis at 30 days; (ii) patients who died between the D30 and M3-visit; (iii) patients for whom the M3-visit was performed much earlier or later than 30 days; (iv) patients with an undated M3-visit; and finally (v) patients with missing data for the EQ-5D-3L®. As shown in Table 5, there was a difference in cumulative average costs at 3 months of € 1,484 (95% CI: 376 to 2,592), with significantly higher average costs for the VATS group (*p*-value = 0.009). There was a difference in the average number of final QALYs of 0.0404 (95% CI: 0.0316 to 0.1123) in favour of the VATS group, but the effect was not significant (*p*-value = 0.2692). This resulted in an ICER of €36,733 per additional QALY gained. The bootstrap analysis resulted in a mean ICER of €324,310 (95% CI:—€273,251 to €921,869). A total of 86.14% of the bootstrapped cost-utility pairs were located in the northeast quadrant of the CU-plane, meaning higher costs and more QALYs following VATS (Fig. 4). The acceptability curve (Fig. 5) revealed a 34% probability of efficiency at 3 months for VATS at a widely-accepted WTP threshold of €25,000 per QALY gained [32]. The results of the cost-utility analysis by multiple imputation (*N* = 254 patients) revealed an increase in the ICER (ICER: €42,779 per QALY) (Table 5), mainly due to a slight increase in the average cost differential after imputation, and the stability in QALY differentials between complete and imputed data.

**Table 5:** Cost-utility analysis, 3 months

		**VATS**^a^			**THORACOTOMY**					
		**N**	**Mean** A	**Median**	**N**	**Mean** B	**Median**	**Difference of means** A-B	***p*-value**	**ICER**^c^ €/QALY
			(SD)	[Q1-Q3]		(SD)	[Q1-Q3]	[95% IC]		
**Complete cases**										
Cumulative costs (€ 2018)		79	11,070	10,849	68	9,586	8,442	**1,484**	0.009	**36,733**
			(3,258)	[8,858–11,844]		(3,536)	[7,467–10,433]	[376–2,592]		
Final QALYs^b^ (year)		79	0.778	0.843	68	0.738	0.824	**0.0404**	0.269	
			(0.206)	[0.664–0.928]		(0,234)	[0.670–0.919]	[0.0316–0.1123]		
Non-parametric bootstrap^e^										324,310
										95% CI: [-273, 251 to 921,869]
**Imputed data**^d^										
Cumulative costs (€ 2018)		129	11,588	10,849	125	9,787	8,442	**1,801**	0.001	**42,779**
			(4, 868)	[8,858–11,872]		(3,622)	[7,467–10,433]	[574–3,199]		
Final QALYs^b^ (year)		129	0.777	0.835	125	0.735	0.810	**0.0421**	0.114	
			(0.196)	[0.673–0.925]		(0.2269)	[0.681–0.894]	[0.0102–0.0944]		
Non-parametric bootstrap^e^										145,067
										95% CI: [26, 291 to 253,843]

^a^Video-Assisted Thoracoscopic Surgery

^b^Quality-Adjusted-Life-Years

^c^Incremental Cost-utility Ratio (noted *ICER*) calculated by relating the difference in mean costs to the difference in mean utilities (QALYs) between the two groups. This ratio is interpreted as the additional cost necessary to gain one additional QALY using the innovative technique (VATS) compared to the reference technique (thoracotomy)

^d^Imputation excluded patients dead and patients who left the study early (lost to follow-up)

^e^Bootstrap is based on 10,000 replications

## Discussion

Of the two strategies, VATS was found to be more expensive, and it did not result in cost reductions in the post-surgical period at 30 days or at 3 months. Like in other RCTs, we did not show significant benefits for VATS in terms of post-operative complications or mortality [16–19]. However, the cost-utility analysis showed that VATS resulted in a slight but significant higher number of QALYs at 30 days after surgery (given a calculated ICER of €19,162/QALY). The bootstrap analysis revealed a 64% probability of efficiency at this time horizon for VATS, at a widely-accepted willingness-to-pay threshold of €25,000/QALY [32] (and only 34% at 3 months).

This trial is the first prospective multicentre RCT to assess the medico-economic impact of VATS compared with open thoracotomy for the management of NSCLC in France. To our knowledge, only one other cost-utility study by Bendixen et al. has been published to date comparing the two strategies [20]. This Danish study was performed in parallel to a clinical RCT between 2008 and 2014, including 103 VATS and 103 thoracotomies. Similar to our study, Bendixen et al. observed significantly better QALYs after VATS at 30 days (+ 0.07 QALY at 4 weeks, *p*-value: 0.008) and higher, but not significantly, QALYs at 3 months (+ 0.02 at 12 weeks, *p*-value = 0.162). As in our study, QALYs were assessed using EQ-5D-3L® and were imputed in case of missing data. However, they found lower overall costs per patient operated by VATS (- €4,267), and 84.3% of the bootstrapped cost-utility pairs were located in the bottom-right quadrant of the CU-plane, meaning lower costs and more QALYs following VATS. The probability of VATS being cost-effective was 95% at DKK 50,000/QALY (€6,720/QALY) according the acceptability curve. However, there were some differences between our studies: theirs was monocentric, designed for the first 12 months following surgery, from the perspective of healthcare services, and included additional costs (consultations with general practitioners, physiotherapists, psychologists, and chiropractors, and prescription drugs). Another more recent publication from 2021 comparing cost and effectiveness between VATS and open lobectomy in China concluded that hospitalization costs were similar for the two strategies, but that there were lower post-surgical costs and higher effectiveness for VATS [13]. However, this study was monocentric and retrospective, and did not present incremental medico-economic ratios. Its results cannot be compared with ours due to differences in defined costs and effectiveness criteria (blood transfusion rate, lung infection rate and post-operative LOS). Regarding the only cost differential between the two strategies, a recent systematic literature review reported that for 19 of 20 studies analysed up to 2020, VATS was associated with higher operative costs [33]. In 17 of them, this cost was significantly counterbalanced by other costs that were lower in VATS compared to thoracotomy during and after discharge. Finally, 10 studies found lower total costs for VATS, 7 found similar total costs, and 3 found higher total costs despite the lower hospitalization costs. Here again, comparisons between these studies and with our results remain difficult. In addition to the fact that the majority of these studies were retrospective and single-centre, the main difference lies in the methodology used for cost evaluation (the choice of perspective, time horizon, expenditure items, and unit costs) and in the characteristics of the included population and the surgeons (when information was available) [33].

The present study has several limitations. Only the hospital perspective was considered insofar as only hospital resources consumed were available based on the data collected in the e-CRF. In this perspective, in order to calculate the production cost per stay as recommended in medico-economic guidelines [28, 30], the DRG were retrospectively determined based on individual information available in the e-CRF, and with the expertise of the medical information department of the Dijon University Hospital. While the initial stays necessarily had the same DRG root (04C02: “*Major surgery on the thorax*”) for which we had only to determine a level of severity (04C021: “*Major surgery on the thorax, level 1”* to 04C024: “*Major surgery on the thorax, level 4”*), those for other re-hospitalisations had to be determined. Moreover, though the production costs per DRG are available in the ENCC, these remain average costs, calculated from 70 representative establishments in France, with the disadvantage of lacking precision on inter-individual variability [34]. However, the ENCC average costs per DRG are recognized as being close to opportunity cost and relevant for economic analyses [34]. The calculation of production costs per stay in the voluntary sample of the 70 hospitals participating in the ENCC follows a common methodology based on the principle of full costs, obtained by allocating expenses to analysis sections and to individual stays according to allocation keys. The national benchmark of the ENCC is presented according to two cost scales, one for public establishments and one for private for-profit establishments. A detailed analysis of costs is available, making them transferable [34]. Then, since the Lungsco01 trial included a micro-costing study based on fifty randomised patients to evaluate the real production cost of VATS and thoracotomy procedures [26], the average surgery cost values obtained were applied to all patients in the trial according to their arm of randomisation, but without adjustment for individual operative time and for individual consumables used. Indeed, the e-CRF was not designed to collect all resources consumed in the operating room for all patients in the trial (except for those included in the micro-costing). However, it is well recognized that a micro-costing study implies a large amount of work that cannot be done on a larger number of patients, but it is the most accurate approach for estimating the real cost of in-hospital healthcare interventions [26, 35]. The fact that the analysis was conducted in the context of a randomized trial allowed comparisons between the two groups and limited potential biases [26]. Another limitation is the number of patients randomized in the study. While the protocol planned to include 600 patients (300 per group), calculated on the basis of the primary clinical endpoint, only 259 patients were finally randomised, demonstrating the major difficulties in carrying out a RCT of this size in thoracic surgery. Another limitation is that the time horizon was 3 months after surgery, not allowing for any potential longer-term effects. However, this can be justified by the fact that neither of the two procedures is likely influence late outcomes (beyond 90 days). Finally, there were missing responses for the EQ-5D-3L® even though it was administered face-to-face. Unfortunately, missing data are a frequent issue in medico-economic analyses within randomised clinical trials. QALYs were thus imputed using the recommended multiple imputation method [36].

Nonetheless, this study has several strengths. This is the first multicentre RCT in France to prospectively evaluate the cost-utility impact of VATS versus open thoracotomy in the management of NSCLC, considering costs from the initial stay and patient follow-up (re-admissions), and utility (QALYs). The centres were required to have performed 50 VATS procedures to be included in the study, thus limiting biases linked to the experience of the surgeons performing VATS. The costing methodology used conforms to the methodological recommendations of health economists [35] and the *Haute Autorité de Santé* (HAS) [28], in terms of *DRG-adjusted* approach for the initial stays and of combination of micro-costing and gross-costing approaches. While micro-costing identifies and measures the resources consumed by the innovative intervention, gross-costing estimates the cost items that can be valued using standard costs (ex: for readmissions related to complications). Finally, our study is one of the few studies that takes ‘utility’ into consideration, though it is a particularly important dimension for medico-economic studies in lung cancer [15, 37–40].

Today, the results of medico-economic studies are considered to be a tool for authorities whose mission is to inform public decision-making on the allocation of health resources (ex: decisions regarding reimbursement). Currently, the procedure of lobectomy or segmentectomy by VATS is valorised through act GFFA009 (*Pulmonary lobectomy, by thoracotomy, with preparation by thoracoscopy*), whose valuation (838.75 €) is insufficient considering the real cost of VATS surgery (3,876.49 €). In our context, the revaluation of VATS should take into account the acceptability threshold of willingness-to-pay that will be set by decision-makers in terms of acceptable supplemental costs per additional QALY gained. Other additional criteria could also be taken into consideration, depending on the decision-making context. For example, multi-criteria decision analysis (MCDA) could also be relevant since it provides information on a wider range of outcomes including physical functioning, psychological well-being, person-centeredness, access to care, and financial affordability [41]. Research comparing MCDA and a cost-utility analysis is needed. Good communication between researchers and decision-makers will be an important part of identifying the most suitable approach [41].

## Conclusions

Given our results, the economic efficiency of VATS at a willingness-to-pay threshold of €25,000/QALY remains fragile at 30 days (64% probability). The economic efficiency is not established beyond that time horizon. However, the acceptability curves given will allow decision-makers to judge the probability of efficiency of this technology depending on other WTP thresholds.

## Data Availability

The clinical datasets generated and/or analysed during the current study are not publicly available because the indirect nominative data cannot be shared publicly under French law, but they can be made available from the corresponding author on reasonable request. The hospital cost data per DRG used during the current study were obtained from the ENCC database (year 2018). Access to this database is freely available on https://www.scansante.fr/applications/enc-mco for the initial stay and re-hospitalisation for complications, and on https://www.scansante.fr/enc-ssr for rehabilitation stays.

## Abbreviations

ARDS: Acute Respiratory Distress Syndrome
ATIH: Agence Technique d’Information Hospitalière (Agency for Information on Hospital Care)
CPI: Consumer Price Index
CRF: Case Report Form
CU: Cost-Utility
CU-plane: Cost-Utility plane
DRG: Diagnosis Related Group
ECOICOP: European Classification of Household Consumption Functions
ENCC: Etude Nationale des Coûts à méthodologie Commune
EQ-5D-3L®: EuroQol 5 Dimensions 3 Levels
ICER: Incremental Cost-Effectiveness Ratio (in context of Cost-Effectiveness Analyses), or: Incremental Cost-Utility Ratio (in context of Cost-Utility Analyses)
LC: Lung Cancer
LOS: Length Of Stay
MCDA: Multi-Criteria Decision Analysis
NSCLC: Non-Small Cell Lung Cancer
QALY: Quality-Adjusted Life Years
RCT: Randomised Controlled Trial
SD: Standard Deviation
VATS: Video-Assisted Thoracoscopic Surgery
WTP: Willingness-To-Pay
